# Breast Tumor-Derived Exosomal MicroRNA-200b-3p Promotes Specific Organ Metastasis Through Regulating CCL2 Expression in Lung Epithelial Cells

**DOI:** 10.3389/fcell.2021.657158

**Published:** 2021-06-24

**Authors:** Pengfei Gu, Mayu Sun, Lei Li, Yang Yang, Zheshun Jiang, Yang Ge, Wenbo Wang, Wei Mu, Hui Wang

**Affiliations:** ^1^State Key Laboratory of Oncogenes and Related Genes, Center for Single-Cell Omics, School of Public Health, Shanghai Jiao Tong University School of Medicine, Shanghai, China; ^2^CAS Key Laboratory of Nutrition, Metabolism and Food Safety, Shanghai Institute of Nutrition and Health, Shanghai, China; ^3^Department of Critical Care Medicine, Ruijin Hospital, Shanghai Jiao Tong University School of Medicine, Shanghai, China; ^4^Department of Oncology, Shanghai Tenths People’s Hospital, School of Medicine, Tongji University, Shanghai, China

**Keywords:** exosomes, organotropic metastasis, tumor immune-suppression, CCL2, microRNA-200b-3p

## Abstract

Malignant metastasis is the most important cause of death in breast cancer (BC) patients, while the lung is a major inflammation and metastatic target organ. Exosomes are nano-sized vesicles that could be uptaken by resident cells to generate the pre-metastatic niche before tumor cells preferentially motility. In the present study, we demonstrated that high expression of C-C motif chemokine ligand 2 (CCL2) in lung could recruit the myeloid-derived suppressor cells (MDSCs) and contribute to the establishment of microenvironment. CCL2 provided recruitment of immune cells under carcinomas conditions and inflammatory responses. We also developed the novel mice model for specific over-expressing CCL2 in the lung, and verified that the BC organotropic metastasis was not because of the enhanced tumor cell proliferation, but the regulatory expression of CCL2 in the target organ. To better explore the crosstalk of exosomal molecules and CCL2 in host tissue, we constructed the “education” lung by exosomes intravenous injection and determined the prominent exosome-uptake by alveolar epithelial type II cells *in vivo*. Furthermore, we identified the exosomal microRNA-200b-3p could bind to *PTEN*, which may involved in the regulation of AKT/NF-κB/CCL2 cascades. Therefore, our study suggest that CCL2 expression in the lung was regulated by BC-derived exosomal microRNA, which primed the pre-metastastatic niche and may be a prognostic marker for the development of BC lung metastasis.

## Graphical Abstract

Schematic diagram of tumor-derived exosomes induced organotropism and immunosuppression.

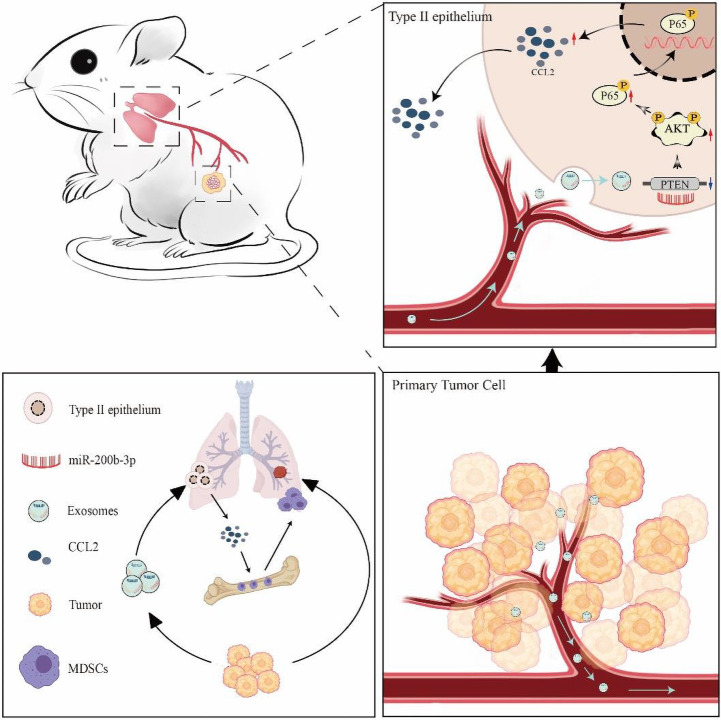

## Introduction

Breast cancer (BC) is the most common type of cancer in women (DeSantis et al., 2011; Woolston, 2015), and approximately 90% of the BC-related deaths are due to recurrence and distant metastasis. Although well-known lungs, bones, and lymph nodes are common metastatic sites of BC, the clinical implications determined that BC lung metastasis uniquely produces symptoms only after the lung sites have been vastly occupied with metastatic tumor masses (Massague and Obenauf, 2016; Tulotta and Ottewell, 2018; Xiong et al., 2018). Therefore, it is of paramount importance for developing the predictive or early diagnostic markers, as well as elucidating the molecular mechanisms in the lung-specificity metastasis. Lung metastatic cascades of BC comprise numerous barriers that must be overcome in order to generate a metastatic preferential microenvironment (Yates et al., 2017). These specific pre-metastatic sites could be stimulated and modulated before primary tumor spread and achieve by diverse components such as growth factor, cytokines, and extracellular matrices (Mu et al., 2019). During these processes, the cytokine C-C motif chemokine ligand 2 (CCL2) plays an extremely important role in target sites through recruiting serious inhibitory immune cells. For instance, CCL2 mediates lung overexpression of endogenous toll-like receptor 4 (TLR4) ligands such as S100A8, which can enhance cancer cell survival in target organ (Yates et al., 2017). The endogenous TLR4-dependent innate immune system plays an important role in pre-metastatic niche formation in the lung, which is an essential procedure of process in lung metastasis. Furthermore, van Deventer et al. also showed that fibroblasts in the blood can recruit myeloid-derived suppressor cells (MDSCs) by secreting CCL2 to construct the lung metastasis-promoting microenvironment, thereby promoting the lung metastasis of melanoma cells (van Deventer et al., 2013). The CCL2/CCR2 axis response the organotropic metastasis of BC due to the recruit of CCL2 in Gr1-positive inflammatory monocytes in the lung (Qian et al., 2011). Although large number of studies have confirmed that CCL2 expression in host organs could promote the metastasis, few study discussed how the primary tumor cells regulate CCL2 expression in these remote tissues before tumor cell motility.

According to the “seed and soil” metastasis hypothesis, primary tumor facility organotropic metastasis through vascular infiltration, spread through the circulatory system, and finally get themselves implanted in remote organs (Fidler, 2003). Among the many factors of metastasis, the adaptation of cancer to the primary tumor microenvironment (TME) and pre-metastasis niche to promote the spread of cancer cells and distant transplantation play vital roles (Costa-Silva et al., 2015; Hoshino et al., 2015). Exosomes are nano-sized vesicles (30–150 nm in diameter) secreted by most cells (Steinbichler et al., 2017; Pegtel and Gould, 2019). They are wrapped in a lipid bilayer and carry various biological molecules, including proteins, glycans, lipids, metabolites, RNA, and DNA. When tumor-derived exosomes are taken up by host cells, these cargoes affect the phenotype of the recipient cell through remodeling the extracellular matrix and stimulating signal transduction of various membrane receptors (Mu et al., 2013). Recently, many clinical pieces of research also indicated that tumor-derived exosomes uptaken by organ-specific cells could prepare the pre-metastatic niche. During these processes, tumor exosomes also could escape from host immune as well as other cancer-related host responses by restricting immune cells. Carrasco-Ramírez et al. (2016) showed that Podoplanin in exosomes promotes the metastasis of melanoma by increasing cell adhesion, cytoskeleton remodeling, and lymphatic vessel formation. Podocalyxin in exosomes also been identified to regulate the transport of integrins in lung fibroblasts, thereby increasing pancreatic adenocarcinoma migration and invasion to target organ (Novo et al., 2018). Exosomes and microRNAs (miRNAs) secreted by cancer cells can be internalized by other cell types in the primary TME and the pre-metastatic/metastatic habitat (Liu et al., 2016). miRNAs are a type of non-coding single-stranded RNA molecule with a length of approximately 18–36 nucleotides encoded by endogenous genes, and they can mediate post-transcriptional gene silencing of target genes by targeting the 3′-UTR of mRNA and further be internalized by other cell types in the primary TME and pre-metastatic/metastatic habitat (Lu and Rothenberg, 2018). Cooks et al. (2018) found that miR-1246-enriched exosomes from colon cancer cells and promote tumor growth and metastasis by reprogramming macrophages into tumor-associated macrophages. Ye et al. (2017) also showed that exosomal miR-141-3p promotes osteoblast activity to promote the osteoblastic metastasis of prostate cancer.

In the present study, we aimed to prove that CCL2 in distal tissues plays a key role in the construction of the microenvironment before tumor metastasis. We have demonstrated that BC cell organotropic metastases to the lung are dependent on the expression of CCL2 in the target organ. We further evaluated and verified that BC-derived exosomes play an essential role in pre-metastatic niche formation before the malignant invasion. BC-derived exosomes contain multiple miRNAs, particularly miR-200b-3p, which is taken up by alveolar epithelial type II cells (AEC II), thereby regulating the microenvironment. Most importantly, we identified that exosomal miR-200b-3p inhibits phosphatase and tensin homolog (*PTEN*), a tumor suppressor, by directly targeting its 3′-UTR. The reduction of *PTEN* further leads to the secretion of CCL2 chemokines, thereby creating a pre-metastasis niche to promote the spread of BC cells to the lungs “Graphical Abstract”. Detailed understanding of the mechanisms underlying BC lung metastasis will shed new light on the identification of novel molecular CCL2 targets to impede daunting pulmonary metastases in patients with BC. Beyond the tumor cell-autonomous view of metastasis, our findings also provide exosomal miRNAs as predict organ-specific biomarkers in BC metastasis.

## Results

### CCL2 Promotes the Growth and Metastasis of BC by Recruiting Myeloid-Derived Suppressor Cells

**FIGURE 1:**
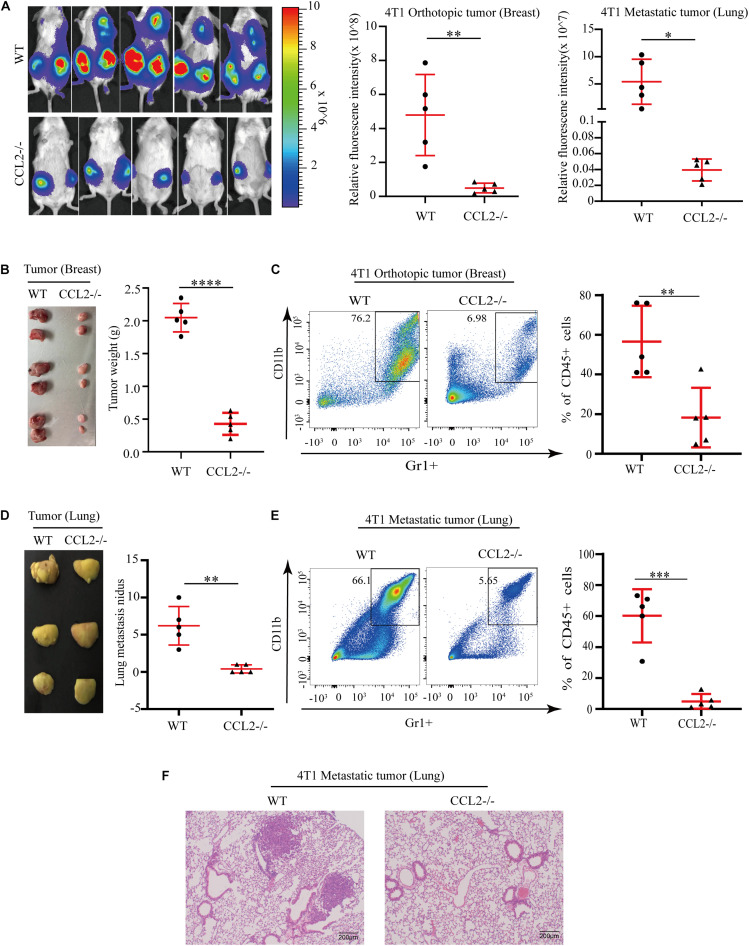
CCL2 promotes the growth and metastasis of breast cancer by recruiting MDSCs. **(A)** Measurement (left) and fluorescence intensity (right) of 4T1 orthotopic growth and lung metastasis in both WT and CCL2/in an *in vivo* imaging system (*n* = 5). **(B)** Representative photographs of orthotopic breast tumor (left) and tumor weight (right) in the mammary gland of WT and CCL2–/– mice after 42 days of 4T1 BC cells transplanted into the mammary fat pad. **(C)** Analysis of tumor infiltration MDSCs in both WT and CCL2–/– mice by FACS (*n* = 5). **(D)** Representative photographs of lung metastasis tumor (left) and lung metastasis nidus counts (right) in both WT and CCL2–/– tumor-bearing mice was counted after fix stained with Bouin’s solution. **(E)** Analysis of MDSCs recruitment in the lung of both WT and CCL2–/– tumor-bearing mice by FACS (*n* = 5). **(F)** Representative hematoxylin-eosin staining of lung metastasis tumor. Bar = 200 μm (magnification: 100×). *P* values were calculated by unpaired Student’s *t*-test (*P < 0.05, **P < 0.01, ***P < 0.001, ****P < 0.0001).

C-C motif chemokine ligand 2 (CCL2) induced various chemokine cascades in the stimulation of target-site tissues and tumor development by enhancing the retention of metastasis-associated immune cells. To verify the effect of CCL2 on both primary tumor proliferation and remote metastasis, 4T1 BC cells were transplanted into the mammary fat pad of wild-type control (WT) and the CCL2 knockout (CCL2−/−) group, respectively. The *in vivo* imaging system was used to determine tumor cell growth and metastasis. Compared with the WT group, the metastatic distribution was significantly reduced in the CCL2−/− group (Figure 1A). Meanwhile, the weight and size of primary breast tumor cells were also evaluated, we found the absence of CCL2 would reduce the more than 4 folds of tumor weight (Figure 1B). CCL2 recruited myeloid-derived CCR2-positive suppressor cells, particularly the MDSCs, that could may active pro-tumorigenic molecules and promote metastasis (Kitamura et al., 2015; Liu et al., 2021). We further analyzed the infiltration of MDSCs in tumor tissues by fluorescence activating cell sorter (FACS) (Figure 1C and Supplementary Figure 1). As shown, the proportion of MDSCs (CD45+/CD11b+/Gr1+) were lower in CCL2−/− mice model after tumor injection, suggesting the CCL2 expression is correlated with BC proliferation and motility.

Besides the primary tumor cell proliferation, we additionally explore the effect of CCL2 in the target organ during the tumor process. Notably, the number of lungs metastatic nidus was evaluated by Bouin’s solution staining (Dong et al., 2017; Hu et al., 2017), they displayed differences between CCL2−/− and WT groups (Figure 1D). The proportion of MDSCs collected from metastasis lung tumor showed a significant decrease in the CCL2−/− group, which suggested a significant contribution of CCL2 to MDSC recruitment to the metastatic site (Figure 1E). Obvious lung metastases were observed in hematoxylin-eosin stained slides of lung cross-sections in WT groups, but not in CCL2−/− group (Figure 1F). Our results shown the expression of CCL2 in host could promote the proliferation and metastasis of BC, meanwhile the blocking of CCL2 contribute to the rescue of spontaneous lung metastasis.

### Specific Expression of CCL2 in the Lung Facilitates BC Metastasis

**FIGURE 2:**
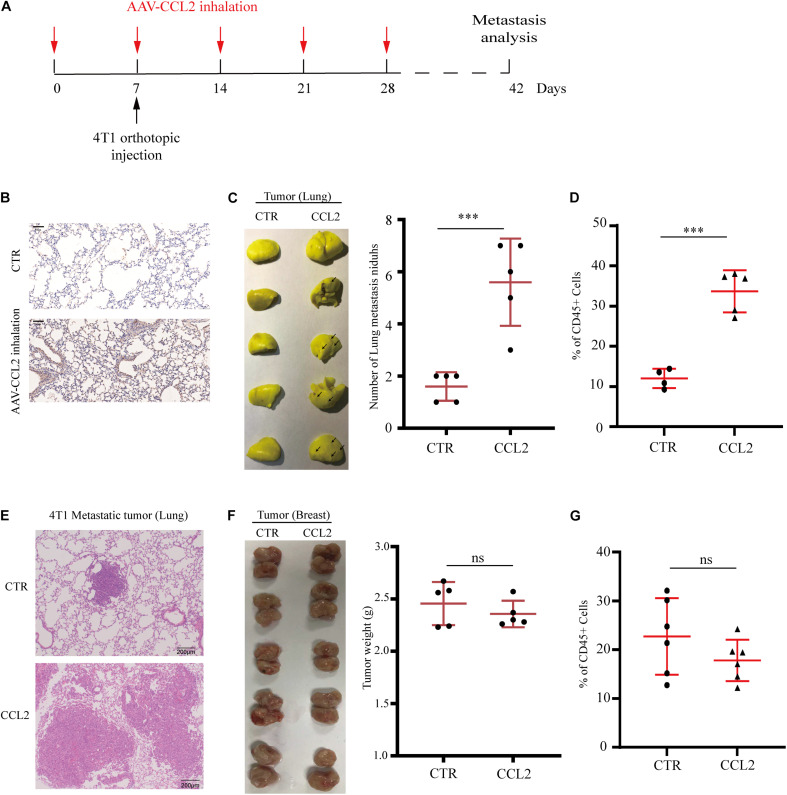
Specific expression of CCL2 in lung facilitates breast cancer metastasis. **(A)** Schematic illustration for the *in vivo* delivery of adenovirus-mediated CCL2 overexpression in lung and spontaneous lung metastasis analysis. **(B)** Representative immunohistochemical staining for CCL2 after AAV-CCL2 inhalation. Bar = 50 μm (magnification: 200×). **(C)** Photography of lung metastasis nidus (left) and lung metastasis nidus counts (right) in both normal and lung-specific CCL2 overexpression tumor-bearing mice was counted after fixed and stained with Bouin’s solution (*n* = 5). **(D)** Analysis of MDSCs recruitment in the metastasis lung tumor of both normal and lung-specific CCL2 overexpression tumor-bearing mice by FACS (*n* = 5). **(E)** Representative hematoxylin-eosin staining of lung metastasis tumor after CCL2 overexpression in lung. Bar = 200 μm (magnification: 100×). **(F)** Photography of orthotopic breast tumor (left) and tumor weight (right) in the mammary gland of normal and lung-specific CCL2 overexpression mice (*n* = 5). **(G)** Analysis of orthotopic breast tumor infiltration MDSCs in both normal and lung-specific CCL2 overexpression mice (*n* = 5). *P* values were calculated by unpaired Student’s *t*-test (***P < 0.001).

Since we have found the expression of CCL2 could enhance local tumor growth *in situ* (Figure 1A), we next wondered whether the organotropic metastasis regulated by CCL2 is mainly because of the target organ modulation rather than tumor cell proliferation. Therefore, to eliminate the pro-metastatic effect of orthotopic tumor proliferation induced by CCL2, a novel mouse model was developed to overexpress CCL2 only in the lung *via* adeno-associated virus (AAV) inhalation (Figure 2A). After 4 weeks of inhalation, the expression of CCL2 in lung increased significantly (Figure 2B), and then 4T1 BC cells were transplanted into the mammary fat pad of mice. We found a dramatic increase in the number of metastatic nidus in the lungs (Figures 2C,E), as well as a significant abundance of MDSCs in the lungs (Figure 2D), but not in primary BC tumors (Figures 2F,G). Therefore, CCL2 expression in the lung had no significant effect on the growth of BC *in situ*, but significantly contribute to the recruitment of MDSCs in the lungs. All these interesting data suggested that the increase of lung metastasis nidus was dependent on CCL2 expression only in the lung instead of stimulating primary growth or generalized expression.

### Breast Tumor-Derived Exosomes Promote Metastasis in a CCL2-Dependent Manner

**FIGURE 3:**
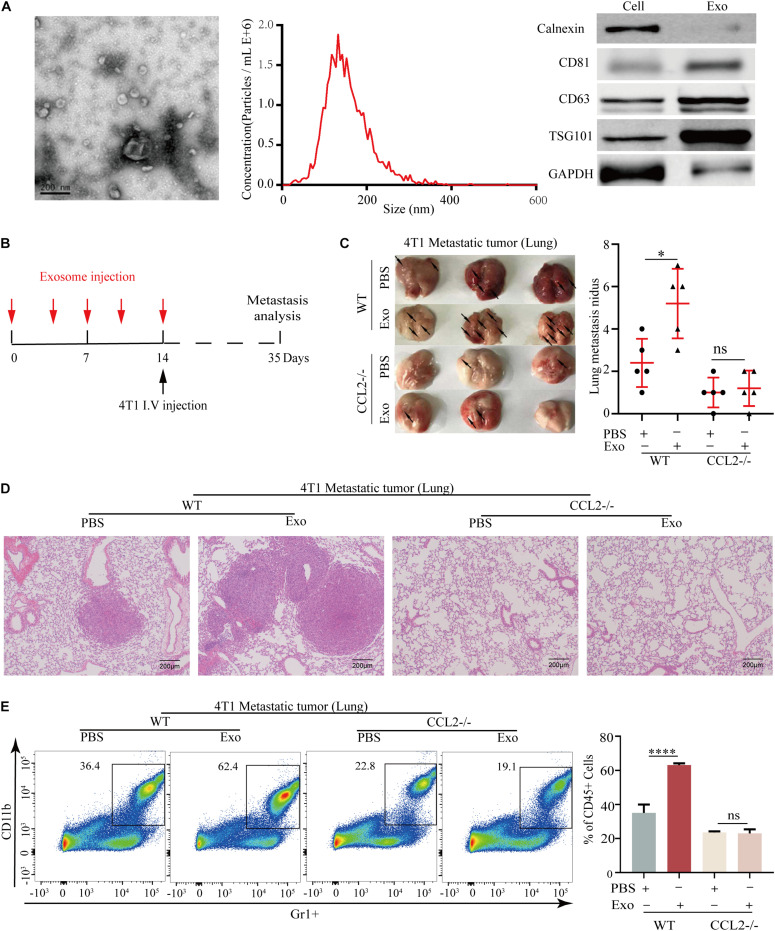
Breast tumor-derived exosomes promote breast cancer metastasis *via* CCL2-dependent manner. **(A)** Electron microscopic identification (left) and nanoparticle tracking analysis (middle) of 4T1 derived exosomes. Western blot analyses of exosomal markers Calnexin, CD63, CD81, TSG101, and GAPDH in 4T1 cells and 4T1 derived exosomes (right). **(B)** Schematic illustration for the *in vivo* 4T1 derived exosomes education and breast cancer lung metastasis analysis. **(C)** Representative photograph of 4T1 lung metastasis tumor (left) and lung metastasis nidus counts (right) in both WT and CCL2–/–mice educated with tumor-derived exosomes or PBS (*n* = 5). **(D)** Representative hematoxylin-eosin staining of lung metastasis tumor in both WT and CCL2–/– mice educated with 4T1 derived exosomes or PBS. Bar = 200 μm (magnification: 100×). **(E)** Analysis of MDSCs recruitment in the lung of both and CCL2–/– mice educated with tumor-derived exosomes or PBS by FACS (*n* = 5). *P* values were calculated by unpaired Student’s *t*-test (*P < 0.05, ****P < 0.0001).

Exosomes secreted by primary tumor cells could generate a pre-metastatic niche by regulating cytokine expression, we herein evaluated the association of BC-derived exosomes and CCL2. The exosomes were collected from the culture supernatant of 4T1 BC cells and confirmed by electron microscope and nanoparticle tracking analysis (NTA) with diameters ranging from 30 to 150 nm. Meanwhile, the exosomal markers (Calnexin, CD81, CD63, and TSG101) were highly expressed in 4T1 derived exosomes by western blot analysis (Figure 3A). For evaluating the effect of BC-derived exosomes *in vivo*, the lung of the animal was modified to provide a metastatic preference niche. Briefly, The 4T1-derived exosomes were intravenously injected into WT and CCL2−/− mice every 3 days to “education” the lung microenvironment, meanwhile the PBS was injected as a control. After 14 days (five times) of education and modulation, we assumed these lungs have been prepared as BC cells preference sites. 4T1 cells were then intravenously injected into exosome-education and control groups, respectively, to determine the effect of exosomes in pulmonary metastasis (Figure 3B). Importantly, we found that compared with CCL2−/− mice, WT mice had significantly more lung metastasis nidus after their lungs were educated with 4T1-derived exosomes (Figures 3C,D). Surprisingly, in CCL2−/− mice, neither PBS control nor exosome-trained group showed BC metastatic nidus, which strongly suggested that promotion of breast tumor-derived exosomes in lung metastasis was dependent on the expression of CCL2 *in vivo* (Figures 3C,D). Consistent with this, the expression of MDSCs in the lung showed a similar trend (Figure 3E), implying that the recruitment of MDSCs was also induced by BC-derived exosomes. The above experiments showed that exosomes secreted by tumor cells could increase the recruitment of MDSCs in the lung, thereby promoting lung metastasis of breast tumor, and this promotion was CCL2-dependent.

### Breast Tumor-Derived Exosomes Regulate Tumor Progression Through the Activation of Alveolar Epithelial Type II Cells

**FIGURE 4:**
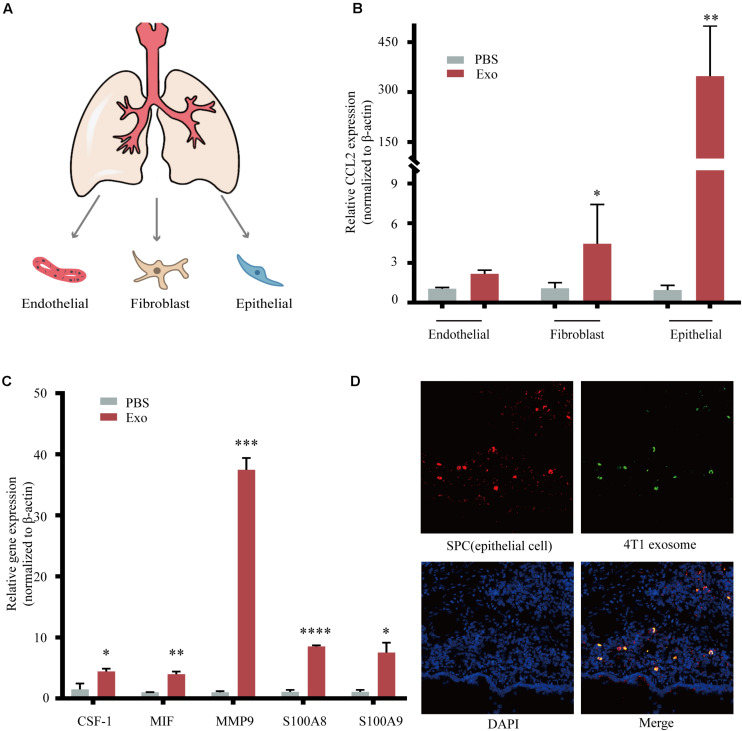
Breast tumor-derived exosomes regulate tumor progression through activation of alveolar epithelial type II cells (AEC II). **(A)** Isolation of primary lung fibroblasts, AEC II, and pulmonary endothelial cell from mouse lung. **(B)** mRNA expression of CCL2 in lung fibroblasts, AEC II, and pulmonary endothelial cells treated with PBS or 4T1 derived exosomes. **(C)** mRNA expression of niche formation associated gene in AEC II treated with PBS or 4T1 derived exosomes. **(D)** Immunofluorescent analysis of the colocalization of tumor-derived exosome and AEC II. *P* values were calculated by unpaired Student’s *t*-test (*P < 0.05, **P < 0.01, ***P < 0.001, ****P < 0.0001).

The metastatic microenvironment can be influenced by both organ-specific factors and the infiltration of different stromal cells. Thus, we determine the three main types of cells including lung fibroblasts, AEC II, and pulmonary endothelial cells that took up exosomes *in vivo* (Nogues et al., 2018; Figure 4A). These three lung cell types were identified by immunofluorescence, using antibodies against Vwf, which identified pulmonary endothelial cells, Vimentin, which identified lung fibroblasts, and SP-C, which identified AEC II (Supplementary Figure 2). After 48 h of co-cultivation with exosomes, we found that the expression of CCL2 in AEC II was significantly increased compared to the other two types (Figure 4B). We also observed upregulation of many key cytokines related with MDSCs recruitment and activation (Figure 4C), including colony-stimulating factor 1 (CSF-1), macrophage migration inhibitory factor (MIF), matrix metallopeptidase 9 (MMP9), and S100 calcium-binding protein A8/9 (S100A8/9) (Kitamura et al., 2015), which are responsible for the recruitment of many inflammatory and immune cells including macrophages and MDSCs, suggesting that exosomes acted on AEC II to build a pre-metastatic niche. Furthermore, co-localization of green fluorescence-labeled exosomes and red fluorescence-labeled AEC II was detected (Figure 4D), indicating that exosomes that passed through the blood and entered the lungs were taken up by AEC II, triggering the above-mentioned series of reactions.

### Exosomal miR-200b-3p Directly Targets *PTEN* to Regulate CCL2 Expression

**FIGURE 5:**
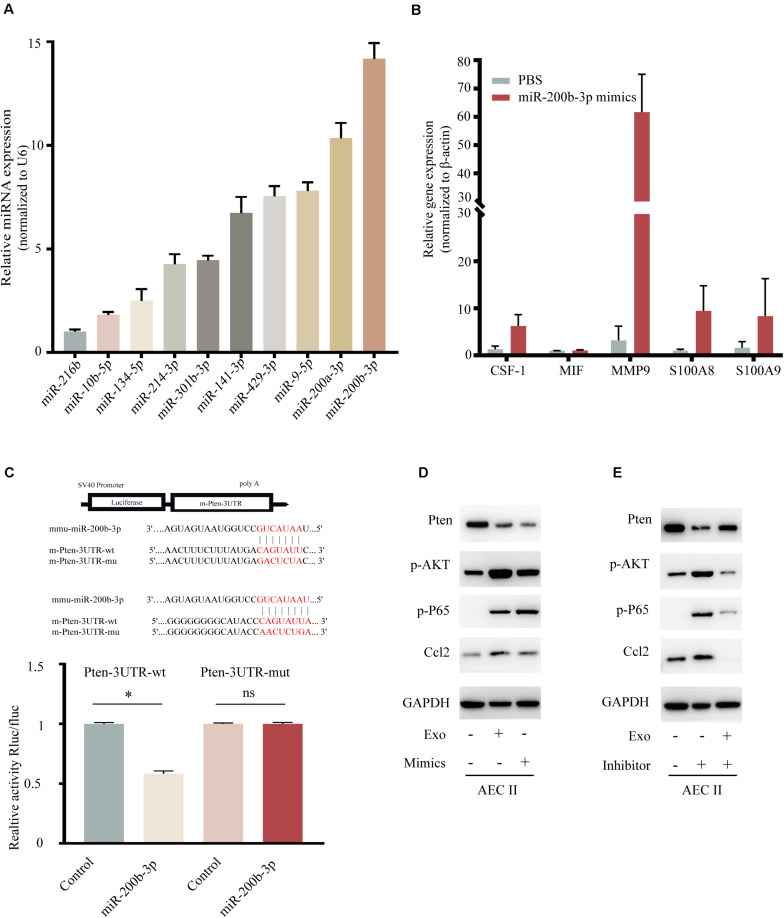
Exosomal miR-200b-3p directly targets *PTEN* to regulate CCL2 expression. **(A)** Relative expression of miRNA in 4T1 derived exosomes. **(B)** mRNA expression of niche formation associated gene in AEC II treated with indicated treatments. **(C)** Dual-luciferase reporter assays showed that miR-200b-3p significantly decreases the relative luciferase activity of the wild-type *PTEN* luciferase plasmid compared with the mutant. **(D)** Western bolt was performed to detect protein levels of *PTEN*, p-AKT, p-P65, and CCL2 in AEC II co-transfected with 4T1-Exo or miR-200b-3p mimics. **(E)** Western bolt was performed to detect protein levels of *PTEN*, p-AKT, p-P65, and CCL2 in exosome-trained AEC II co-transfected with miR-200b-3p inhibitors. *P* values were calculated by unpaired Student’s *t*-test (*P < 0.05).

Recently, various miRNAs including miR-216b, miR-10b, miR-134, miR-214, miR-301b, miR-141, miR-429, miR-9, and miR-200a/b were demonstrated to involved in BC proliferation and motility (Bach et al., 2017; Zhang et al., 2018; Sueta et al., 2019; Wortzel et al., 2019), but the mechanism for lung-specific metastasis still need exploration. We evaluated the expression of several miRNAs in 4T1-derived exosomes, as shown in Figure 5A, miR-200b-3p was highly expressed in exosomes. Additionally, the expression of CSF-1, MIF, MMP9, and S100A8/9 also showed the trend in the mimic of the miR-200b-3p group (Figure 5B) comparing with exosome-modulation groups (Figure 4C). The binding sites between miR-200b-3p and the 3′-UTR of mRNAs were predicted using TargetScan, and we identified *PTEN* as the miRNA target because of the well-characterized role of this gene in tumor biology (Hopkins et al., 2014; Worby and Dixon, 2014; Alvarez-Garcia et al., 2019). A dual-luciferase reporter assay was employed to determine whether *PTEN* was a direct target of miR-200b-3p. Luciferase activity was significantly reduced following co-transfection with miR-200b-3p and WT *PTEN* 3′-UTR compared with the control; however, luciferase activity was not altered following co-transfection with miR-200b-3p and mutant *PTEN* 3′-UTR (Figure 5C). These results provided direct evidence that *PTEN* was a target of miR-200b-3p. Results demonstrated that the expression of *PTEN* decreased significantly, while the phosphorylated AKT, phosphorylated p65, and CCL2 were all upregulated in AEC II co-cultured with 4T1-derived exosomes or those co-transfected with miR-200b-3p mimics (Figure 5D and Supplementary Figure 3A). In addition, the opposite conclusion was obtained after transfection of exosome-trained AEC II cells with miR-200b-3p inhibitor (Figure 5E and Supplementary Figure 3B). These observations implicated *PTEN*-dependent activation of AKT/NF-κBp65 signaling, which promoted CCL2 expression in AEC II.

## Discussion

In this study, we demonstrated the BC-derived exosomes could increase the expression of CCL2 in the target organ, thereby contributing to a generation of the immune-suppression microenvironment (increased levels of MDSCs) in the lung and promote lung-specific metastasis. We indicated that highly expressed mir-200b-3p delivered by BC exosomes were taken up by AEC II, the target cells in the lung, and directly target to *PTEN*. Inhibition of *PTEN* further promotes the activation of the AKT/NF-κBp65 pathway, which ultimately increases the expression of CCL2. Moreover, CCL2 recruits CD11b^+^Gr-1^+^ cells (MDSCs) in the target organ microenvironment to develop a pre-metastasis niche for BC metastasis. In the present study, we established a novel animal model to overexpress CCL2 in mouse lungs to verify the effect of the expression of CCL2 in the target organ during BC metastasis. Our experimental results showed that overexpression of CCL2 in the lungs could indeed increase the recruitment of MDSCs, thereby promoting lung metastasis of BC. However, the overexpression of CCL2 in the lung had no significant effect on the growth of BC tumors *in situ*. This suggested that in the process of lung metastasis of BC, lung CCL2 could become a powerful tool for constructing a metastatic microenvironment.

Distant metastasis accounts for the vast majority of deaths in BC patients who normally exhibit the distinct metastatic patterns involving bone, liver, lung, and brain. BC can be divided into different subtypes based on gene expression profiles, and different BC subtypes show preference to distinct organ sites of metastasis (Weigelt et al., 2005). The basal-like breast cancer (BLBC) displays a lung tropism of metastasis which contributed to a low life expectancy (Jin et al., 2018). In particular, 60–70% of metastatic BC patients who eventually died were diagnosed with lung metastasis (Chen et al., 2019). Therefore, underlying mechanisms of the organ-specific pattern in BC metastasis remain to be elucidated. Extensive experimental models confirmed the “’seed and soil” theory, which indicates that tumor cells (seed) grow preferentially in selected organs (soil) (Langley and Fidler, 2011; Akhtar et al., 2019). Under normal circumstances, the growth environment of remote organs and tumors *in situ* is very different, which makes the remote organs unsuitable for the growth of metastatic tumors. However, under various pressures, such as hypoxia and nutritional deficiencies, tumor cells activate various metastasis-promoting mechanisms to help them successfully colonize and grow in remote tissues (Sceneay et al., 2013). The most important mechanism involves vesicles, cytokines, and growth factors secreted by tumors *in situ*, which recruit bone marrow-derived cells in remote organs to help them build a pre-metastatic niche. Furthermore, Qian et al. (2011) also have demonstrated that CCL2 neutralization attenuates the recruitment of inflammatory monocytes and reduces metastasis in breast tumor-bearing mice. In the present research, we used CCL2 knockout mice to construct a spontaneous BC metastasis model, and compared with that of normal mice, the proportion of MDSCs recruited in the lungs of tumor-bearing mice was significantly reduced, and the lung metastasis was also greatly reduced. We showed that CCL2 was essential for tumor metastasis as it recruits MDSCs to build a microenvironment that promoted metastasis, which was consistent with the results of previous studies. It is worth mentioning that our research results also showed that BC growth *in situ* was significantly reduced upon CCL2 knockout in mice. We observed that the proportion of MDSCs in breast tumors was also reduced *in situ*, indicating that CCL2 promoted tumor growth *in situ*. However, there was no direct evidence that CCL2 could directly promote lung metastasis by recruiting such cells in the primary breast tumor. These areas of feasibility can be explored in the future.

Breast cancer cells can use CCL2 in remote organs to recruit MDSCs to build a pre-metastasis niche and promote subsequent metastasis. However, how distant tumor cells use the CCL2 expression in the lung is still unclear. Our research revealed that after the tumor-derived exosomes reach the lungs in normal mice, they could cause more MDSCs to accumulate in the lungs, and ultimately promote lung metastasis, which was consistent with previous studies. However, we also found that in CCL2 knockout mice, tumor-originated derived exosomes did not play the same role, which indicated that BC-derived exosomes used CCL2 to construct the pre-metastasis niche. In addition, we conducted the two tumor originals exosomes to determine the effect of exosomes in target cell uptaken. Besides using 4T1-derived exosomes, another exosomes from the syngeneic weakly metastatic cell line, 4TO7, was collected to show the lung metastasis. Although both the 4TO7 and 4T1 exosomes could be taken up by lung epithelial cells, the 4T1-derived exosomes increased CCL2 expression, which may occur due to the expression of exosomal miR-200 or other unknown cytokines in exosomes (Supplementary Figure 4). Identified ACE II as target cells in the lungs. ACE II accounts for 60% of lung epithelial cells and plays a key role in immune processes, such as lung tumor metastasis (Sceneay et al., 2013; Liu et al., 2016). Therefore, exosomes secreted by BC cells can construct a metastasis-promoting microenvironment through ACE II. More importantly, we found that the marker gene expression of the pre-metastasis niche in ACE II stimulated by exosomes was also significantly increased. Therefore, we speculated that exosome-mediated CCL2 might promote metastasis by recruiting MDSCs to interact with lung stromal cells to further construct the pre-metastasis niche and the upregulation of cytokines related with MDSCs recruitment and activation also proved this point. miRNA, the main component of cancer exosomes, can mediate the efficient and rapid silencing of mRNAs in target cells to reprogram the target cell transcriptome. For instance, exosomal miR-1247-3p in hepatocellular carcinoma cell-derived cells can target cancer-associated fibroblasts, promoting hepatocellular carcinoma metastasis (Fang et al., 2018). *PTEN* is a negative regulator of PI-3 kinase-dependent signaling (Harrington et al., 2005; Haddadi et al., 2018; Papa and Pandolfi, 2019). Activation of PI-3 kinase results in the activation of AKT and downstream mediators involved in cell survival, such as the mammalian target of rapamycin (Porta et al., 2014). Previous studies have also shown that downstream signals of PI3K/AKT/NF-κBp65 increase CCL2 expression. For example, brain astrocyte-derived exosomes can promote the outgrowth of brain metastatic cancer cells by transferring *PTEN*-targeting miR-19a to the target tissue (Aleckovic and Kang, 2015). Although the implementation of BC screening programs and improved options for the treatment of patients with early BC have contributed to the improved outcome in BC, once the metastatic disease develops, BC remains highly lethal. Exploring the organ-specific manner of primary BC could help to improve the modalities for the treatment of the primary tumor and of metastatic disease.

## Materials and Methods

### Cell Line

The mouse BC cell line, 4T1, was purchased from the Cell Bank of Shanghai Institute of Cell Biology, Chinese Academy of Sciences (Shanghai, China) and cultured in Dulbecco’s modified Eagle’s medium (DMEM) with 10% fetal bovine serum (Hyclone) and 1% penicillin/streptomycin (Invitrogen). Mouse primary pulmonary endothelial cells, lung fibroblasts, and AEC II were isolated from Balb/c and cultured respectively in endothelial cell medium (Science), DMEM medium, and alveolar epithelial cell medium (ScienCell). All cells were cultured in a 5% carbon dioxide incubator at 37°C.

### Labeling of Cells With Luciferase

4T1 cells were seeded in 6-well plates. When cells grew to 50–60% confluence, they were infected with the lentivirus overexpressing mCherry in the presence of 10 μg/mL Polybrene (Sigma) for 4–6 h. Then 48 h later, the infection efficiency was observed with an inverted fluorescence microscope and the positive cells with red fluorescence were sorted by flow cytometry.

### Antibodies and Reagents

Mouse flow cytometry antibodies CD16/CD32 (553141), CD45 APC-Cy7 (557659), CD11b PE (553311), Ly-6G and Ly-6C FITC (553127), and flow cytometry buffer (Stain Buffer FBS) were purchased from BD Pharmingen (United States). The red blood cell lysate was purchased from Biyuntian Company (China). Type II lung epithelial cell isolation and identification antibody Biotinylated anti-mouse CD45, Biotinylated anti-mouse TER119, Anti-mouse EpCAM-APC were purchased from eBioscience (United States). Biotinylated anti-mouse Integrin β4, Biotinylated anti-mouse CD31 were purchased from Biolegend (United States). Streptavidin-PE, Biotinylated anti-mouse CD16/32 were purchased from BD Pharmingen (United States). Dynabeads^®^ MyOne^TM^ streptavidin T1 magnetic beads, Rabbit anti-pro-SP-C, and DAPI were purchased from Thermo Fisher Scientific (United States). Dispase II, low melting point agarose gel, DNase I were purchased from Sigma (United States). VWF, VWFpp polyclonal antibody and vimentin polyclonal antibody were purchased from Proteintech (United States). Mcp1 Monoclonal antibody was purchased from Proteintech (United States). Mouse antibodies against CD63, Tsg101, and CD81 were purchased from Santa Cruz Biotechnology (Santa Cruz, CA, United States). Rabbit antibodies against PTEN, Phospho-NF-κB p65 Phospho-Akt, Calnexin were purchased from Cell Signaling Technology (MA, United States).

### Isolation of Different Cell Types

Four-week-old BALB/c mice were killed by administration of an anesthetic overdose (0.5 mL/10 g 5% chloral hydrate). The mouse primary pulmonary endothelial cells, lung fibroblasts, and AEC II were isolated as below:

Isolation of lung fibroblasts: (a) take an appropriate amount of lung tissue, remove the trachea in the lung, (b) cut into pieces, and wash at least three times, (c) digest with 0.1% I, II, and IV collagenase and 0.1% dispase at 4°C overnight, (d) then go through 100 mesh Sieve, (e) take the filtrate, centrifuge at 300*g* for 5 min, and (f) resuspend and spread the bottle.

Isolation of pulmonary endothelial cells: (a) Take the edge of the lung and cut it into small pieces. (b) Move the tissue block into a T25 culture flask and place it upside down in the cell culture incubator. (c) After 2 h, inject 2 mL of endothelial culture medium into the culture flask and place the culture flask carefully. Incubate the incubator to ensure that the tissue block does not float up. (d) Change the medium to 2 mL every 3 days. After the cells crawl out of the tissue block, change the medium every 2 days. When the cell fusion reaches about 60–70%, remove the tissue block. Pulmonary microvascular endothelial cells were re-plated in the flask.

Isolation of AEC II: (a) Take an appropriate amount of lung tissue and remove the trachea in the lungs. (b) Cut into pieces and wash at least three times. (c) Digest with 0.25% Trypsin at 37°C for 15 min, then add a serum to stop, (d) digest with 0.1% type I collagenase for 1.5–2 h or so, (e) pass through a 100-mesh screen, (f) take the filtrate, centrifuge at 300*g* for 5 min, and (g) resuspend, and spread the bottle.

### Immunofluorescence

Cells grown on cover-slips were fixed with 4% formaldehyde in PBS for 15 min at room temperature, then washed three times with PBS. Membrane breaking solution was then added for 30 min, and washed three times with PBS. The first antibodies diluted as appropriate were applied to the slides and incubated overnight at 4°C. Following three washes with PBS, secondary antibodies conjugated with fluorescence were added, and slides were incubated at room temperature for 2 h while avoiding light. After washing with PBS for 10 min, we added DAPI dye solution for 5–10 min, covered with a glass slide, and then sealed.

### HE Staining and Immunohistochemical Staining

HE staining: the tissue sections were stained with hematoxylin for 5 min, then soaked in 1% acid ethanol (1% hydrochloric acid 70% ethanol), and then rinsed with distilled water. The sections were dehydrated with 3 min, alcohol by eosin staining and clearing by xylene. Then use the Olympus BX53 fluorescence microscope (Tokyo, Japan) to examine and photograph. Immunohistochemical staining is conducted follow the protocol of the Immunohistochemistry Kit (Sango Biotech, Shanghai, China).

### Isolation and Purification of Exosomes

Exosomes were purified by differential centrifugation processes. Exosomes were prepared as follows: supernatant was collected from cells that were cultured in media without FBS for 24 h and were subsequently subjected to sequential centrifugation steps for 800*g* for 5 min, and 2,000*g* for 10 min. This resulting supernatant was then filtered using 0.2 μm filters, and a pellet was recovered at 100,000*g* in an SW 32 Ti rotor after 2 h of ultracentrifugation (Beckman). The supernatant was aspirated and the pellet was resuspended in PBS and subsequently ultra-centrifuged at 100,000*g* for another 2 h. The purified exosomes were then analyzed and used for experimental procedures.

### Quantification, Identification, and Staining of Exosomes

The protein concentration of aliquots (25 and 50 μl per sample) of each lysate was determined in a 96-well plate against HSA standards in PBS (ranging from 1 to 10 μg), applying the Bradford test kit by Bio-Rad, and the total amount of proteins per well was calculated. Exosomes were labeled with fluorescent dye the PKH-67 labeling kit. For exosome co-cultures experiment, 200 ug/mL of 4T1-derived exosomes were incubated with the AEC II (6 × 10^5^) in the exosomes depleted culture medium.

### Construction of Mouse Model

Construction of an orthotopic model of mouse BC: The mice were anesthetized with an intraperitoneal injection of 4% chloral hydrate, and fixed on the mouse plate after anesthesia. A total of 1 × 10^5^ 4T1 cells stably expressing firefly luciferase with matrix gel were injected into the both-sides fourth mammary gland fat pad. After the injection, the wound was sutured. Subsequent weekly live imaging was used to detect the growth of tumor *in situ* and lung metastasis by the Xenogen IVIS imaging system (Perkin-Elmer, Fremont, CA, United States). Mice were anesthetized with 2% isflurane and imaged 5 min after injection with 150 mg/mL D-luciferin (Alameda, CA, United States) intraperitoneally. Mouse model of exosomal “education”: each mouse received tail vein injection of 10 μg exosomes (dilute with 100 μl PBS, exosomes derived from 4T1 cells) five times in 2 weeks.

### Flow Cytometric Analysis

To obtain single-cell suspensions of tumors, the tissue was digested using a mouse tumor digestion kit and Gentle MACS instrument (Miltenyi Biotec) according to manufacturer instructions. Red blood cells were removed using an RBC lysis buffer. Cells were added to a 96-well plate U-bottom shape and stained with live/dead aqua stain for 30 min in darkness. Then cells were blocked using an anti-mouse CD16/CD32 antibody. For antibody staining, cells were washed twice in sorting buffer (1% FBS/PBS), before incubation in antibody (diluted in sorting buffer) on ice and in the dark, for 30 min. All samples were assayed using a Beckman Gallios Flow Cytometer. Data were analyzed by FlowJo analysis software.

### Western Blot

The cells were cleaved by the cell lysate containing protease and phosphatase inhibitors, and quantified by Bradford method. The quantitative protein 35 μg was taken for SDS-PAGE electrophoresis. After the electrophoresis was over, the protein was transferred to the PVDF membrane. A total of 5% skim milk was closed for 1 h and washed the membrane three times for 10 min each time. The first antibody was diluted with 5% bovine serum albumin and incubated overnight at 4°C. PBST rinse the film three times for 10 min each time. The second antibody was diluted with 5% bovine serum albumin and incubated for 1 h, then PBST washed the membrane three times for 10 min each time. The protein expression was detected by chemiluminescence reagent combined with imager.

### Real-Time Quantitative PCR

The cell was lysed by Trizol (Ambion) lysate, and the cell RNA was extracted by Direct-zol-96 MagBead RNA kit (ZYMO Research), and the concentration of the extracted RNA was determined and the quality was controlled. The primers of related genes were designed by PrimeScript RT reagent Kit (Takara) reverse transcription for cDNA, and real-time PCR detection was carried out by using SYBR Premix EX Taq (Takara) in 7900HT Fast Real-Time PCR System (Applied Biosystems). The relative expression was calculated by ΔΔCt method.

### Dual-Luciferase Reporter Gene Assay

The binding site between miR-xxx and xxx 3′-untranslated regions (3′UTR) was predicted by TargetScan database version 7.1^1^. Wild-type (wt) and mutant type (mut) XX 3′UTR were separately inserted into pMIR-REPORT vectors (Ambion, Austin, TX, United States). Vectors and miR-200b-3p mimics or negative control were co-transfected into HEK-293T cells, then luciferase activity was detected using the Dual-Luciferase Reporter Assay System (Promega, Madison, WI, United States).

### Overexpression of CCL2 by Adeno-Associated Virus

The target gene CCL2 (GenBank ID:NM_011333-P2A) and its upstream and downstream sequences were searched from GenBank, and the primers were designed with VectorNTI software. Linearized expression vector (AOV002:pAAV-CAG-MCS-EGFP-3FLAG) was purchased from OBiO Technology (Shanghai) Corp. The target gene fragment was recombined with the linearized vector by Geneart seamless cloning and assembly kit (Thermo Fisher Scientific). The target gene fragment was ligated with the linearized vector by T4 DNA Ligase. Ligation mix was transformed into competent DH5 alpha cells, and positive colonies were selected and enlarge cultured for plasmid. Colony PCR was performed to screen for positive clones which were used for plasmid extraction by using a plasmid miniprep kit (Qiagen), and the resultant DNA was sent for sequencing (forward sequencing primers: CAG-F AGAGCCTCTGCTAACCATG; reverse sequencing primers: pEGFP-N-3 CGTCGCCGTCCAGCTCGACCAG). Plasmid was introduced into 293 AAV cells to generate viruses for overexpression of CCL2.

### Statistical Analysis of Experimental Data

GraphPad Prism software was used for data analysis and mapping. All *in vitro* and *in vivo* experimental results were expressed as the mean ± standard error of mean (SEM) of at least three independent experiments and analyzed with Student’s *t*-test (two-tailed). *P* < 0.05 was considered to indicate a statistically significant difference.

## Data Availability Statement

The raw data supporting the conclusions of this article will be made available by the authors, without undue reservation.

## Ethics Statement

The animal study was reviewed and approved by the Institutional Animal Care and Use Committee (IACUC) of the Institute for Nutritional Sciences. Written informed consent was obtained from the owners for the participation of their animals in this study.

## Author Contributions

HW and WM: conceptualization. MS and PG: methodology, validation, formal analysis, and visualization. YY: software. ZJ, WW, and LL: investigation. HW: resources. PG: data curation and writing—original draft preparation. WM: writing—review and editing. WM and HW: supervision and project administration. HW, WM, and YG: funding acquisition. All authors have read and agreed to the published version of the manuscript.

## Conflict of Interest

The authors declare that the research was conducted in the absence of any commercial or financial relationships that could be construed as a potential conflict of interest.

## Footnotes

1 http://www.targetscan.org/vert_71

## Supplementary Material

Supplementary Figure 1 Gating strategies for the identification of MDSCs subsets. Gating strategy used to define MDSC subpopulations in orthotopic breast tumor (above) and lung metastasis tumor (below).

Supplementary Figure 2 Immunofluorescence analysis for primary lung cells. Immunofluorescence analysis for pulmonary endothelial cells, lung fibroblasts and alveolar epithelial type II cells.

Supplementary Figure 3 Quantification of the protein expression. **(A)** Quantification of the relative expression of the proteins in AEC II co-transfected with 4T1-Exo or miR-200b-3p mimics. **(B)** Quantification of the relative expression of the proteins in exosome-trained AEC II co-transfected with miR-200b-3p inhibitors.

Supplementary Figure 4 Relative expression of the proteins. mRNA expression of CCL2 in lung fibroblasts, AEC II, and pulmonary endothelial cells treated with 4TO7 or 4T1 derived exosomes.
