# Supplementary figures and images for: Breast Tumor-Derived Exosomal MicroRNA-200b-3p Promotes Specific Organ Metastasis Through Regulating CCL2 Expression in Lung Epithelial Cells
Source: Front Cell Dev Biol. 2021 Jun 24;9:657158. doi: 10.3389/fcell.2021.657158 (PMC8264457; doi:10.3389/fcell.2021.657158)

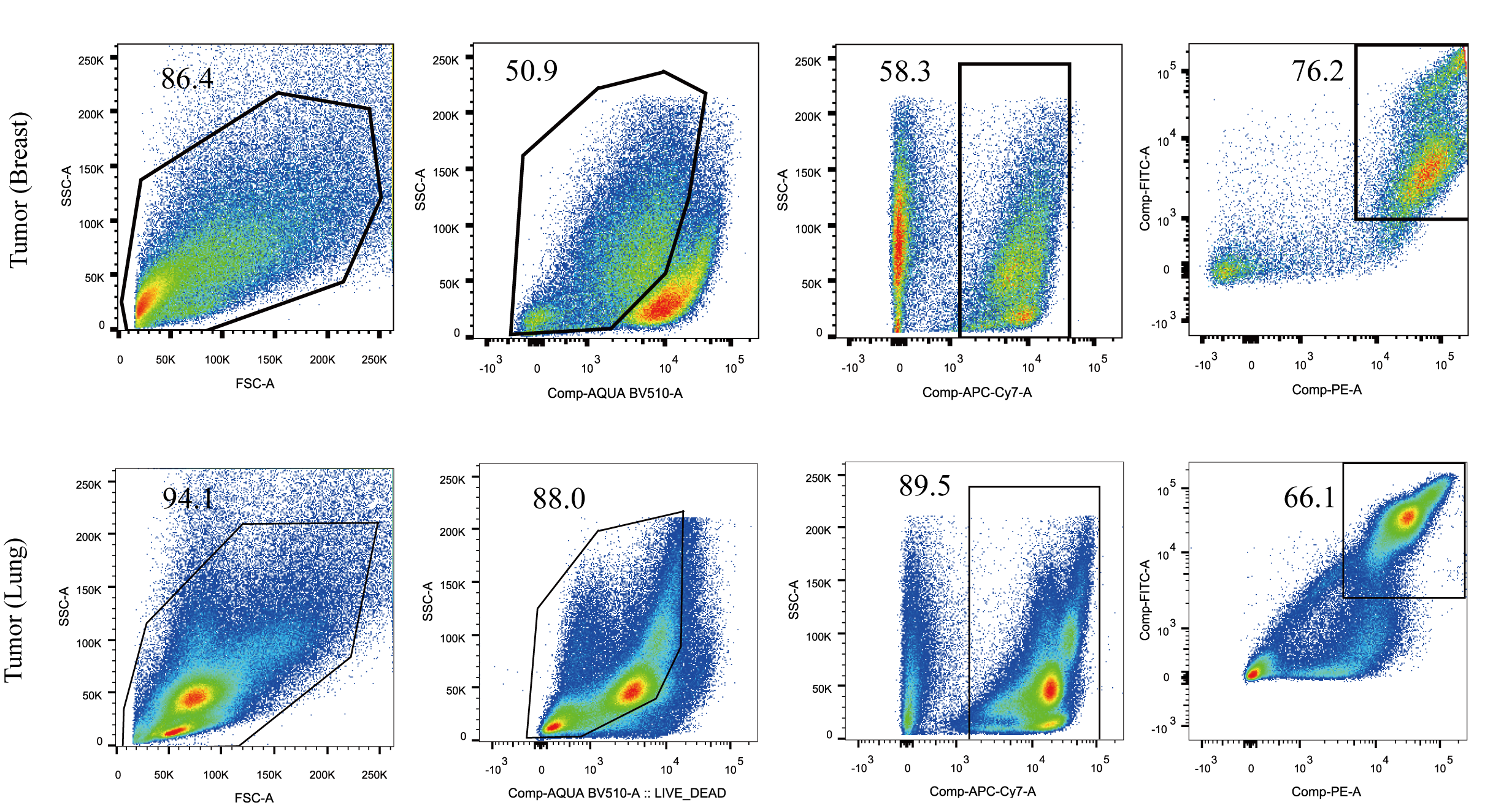

Supplement: Supplementary Figure 1 — Gating strategies for the identification of MDSCs subsets. Gating strategy used to define MDSC subpopulations in orthotopic breast tumor (above) and lung metastasis tumor (below). [file Image_1.tif]

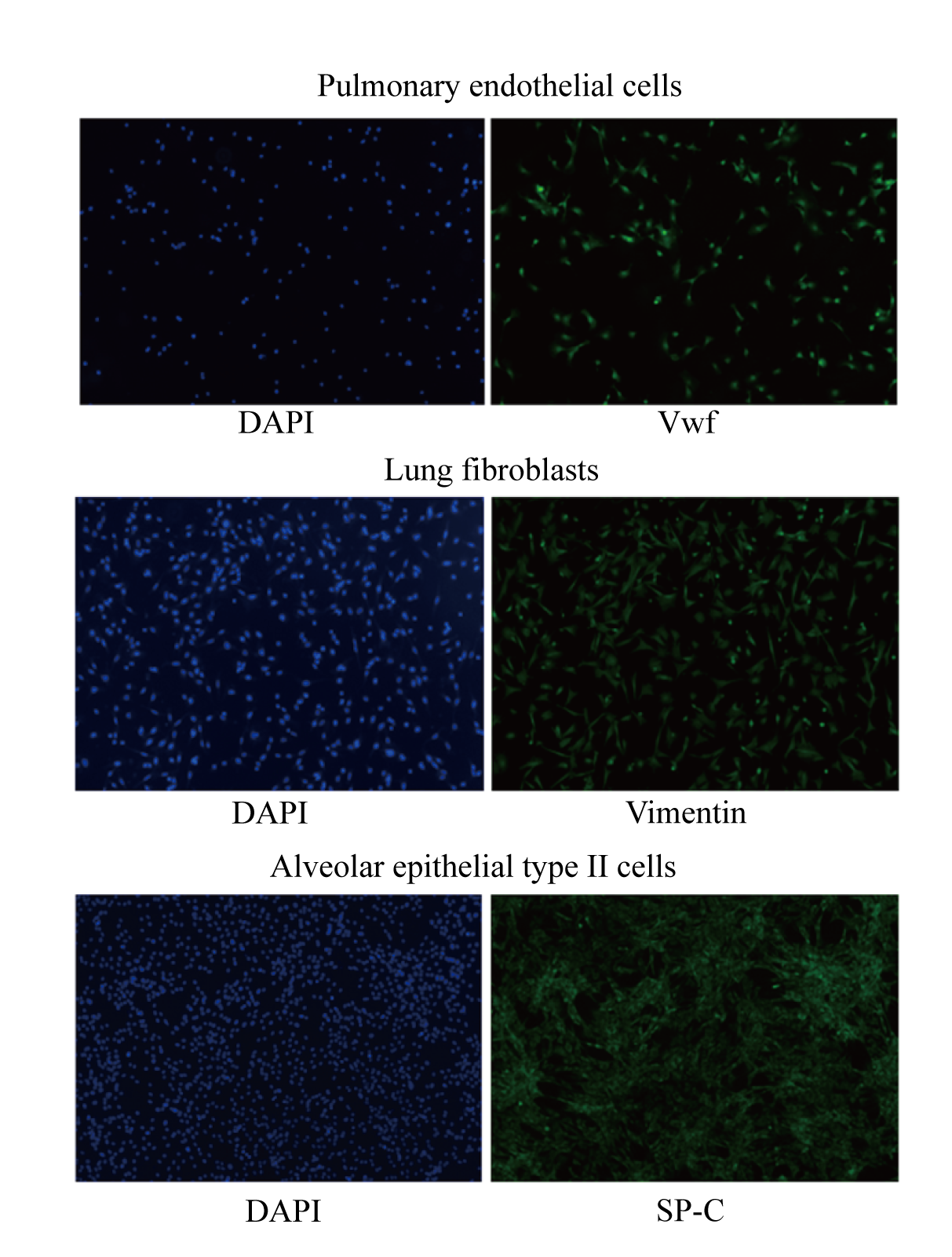

Supplement: Supplementary Figure 2 — Immunofluorescence analysis for primary lung cells. Immunofluorescence analysis for pulmonary endothelial cells, lung fibroblasts and alveolar epithelial type II cells. [file Image_2.tif]

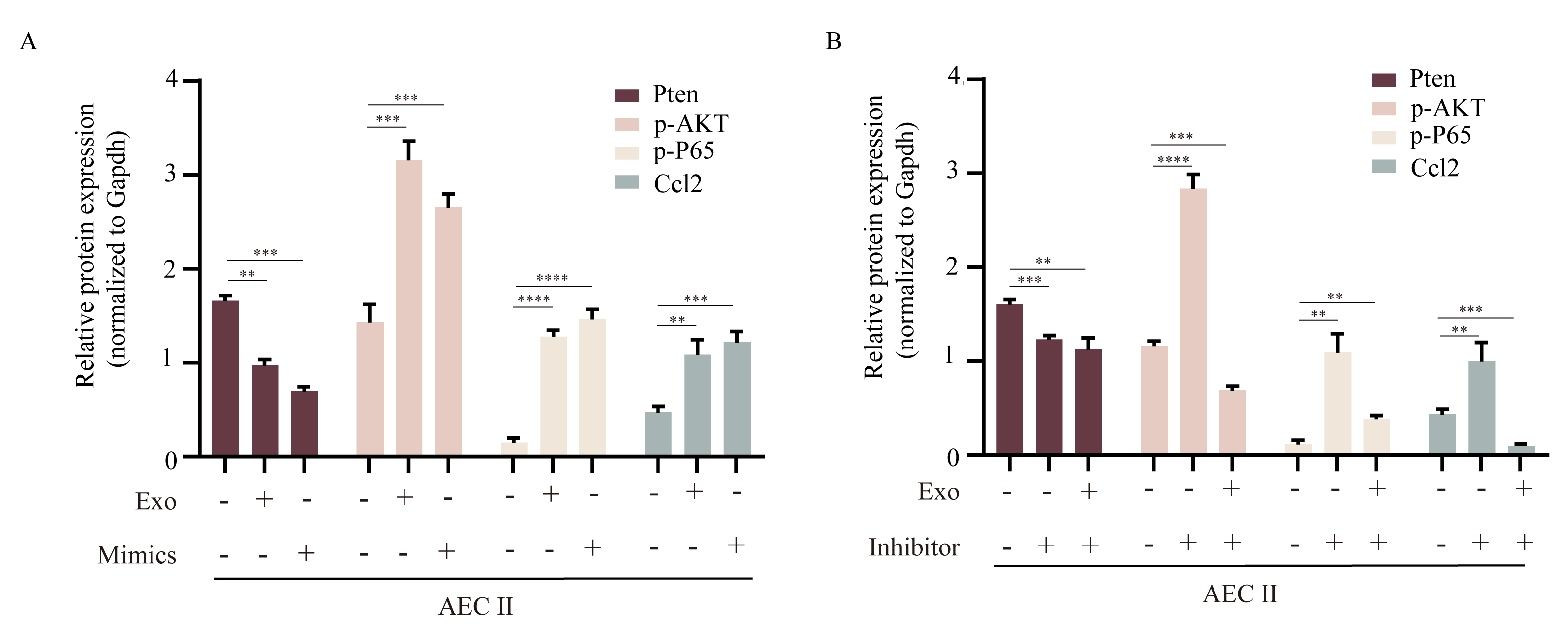

Supplement: Supplementary Figure 3 — Quantification of the protein expression. (A) Quantification of the relative expression of the proteins in AEC II co-transfected with 4T1-Exo or miR-200b-3p mimics. (B) Quantification of the relative expression of the proteins in exosome-trained AEC II co-transfected with miR-200b-3p inhibitors. [file Image_3.tif]

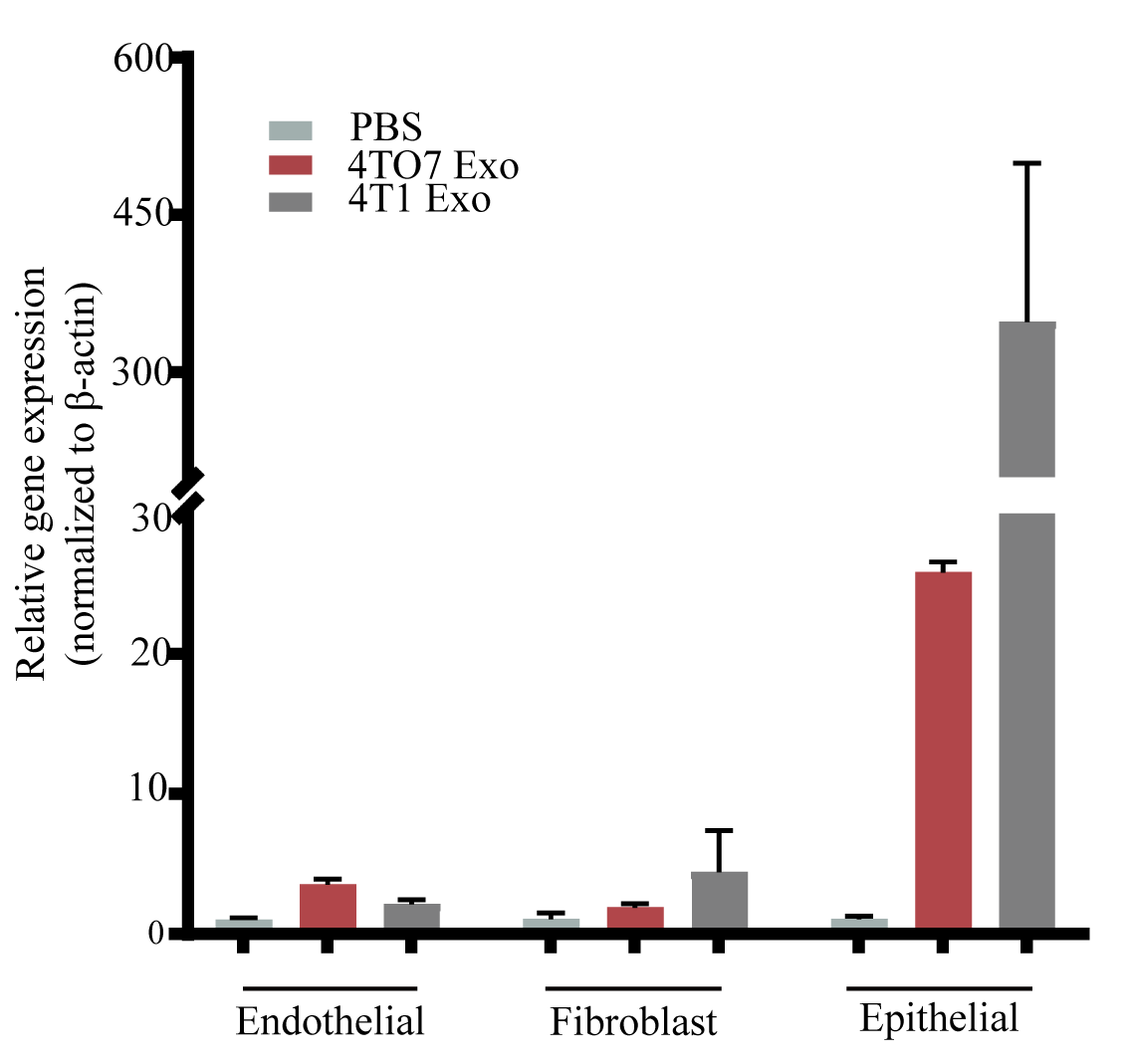

Supplement: Supplementary Figure 4 — Relative expression of the proteins. mRNA expression of CCL2 in lung fibroblasts, AEC II, and pulmonary endothelial cells treated with 4TO7 or 4T1 derived exosomes. [file Image_4.tif]
